# The Antitoxin Treatment of Hay Fever

**Published:** 1903-08-01

**Authors:** 


### Hospital Clinics and Medical Progress.

### THE ANTITOXIN TREATMENT OF HAY FEVER.

Sir Felix Semon has recently published[1] some interesting impressions formed from the treatment of eight cases of hay fever with Professor Dunbar's antitoxin. The word "impression" is his own, and he has adopted it, he says, because he has never before in any scientific inquiry found such difficulty in satisfying himself as to what conclusion he should form. He was prepared to find that the effect of the remedy would be variable in intensity in different hay-fever patients, but the differences actually found were so great that it is a matter of difficulty for a conscientious observer to briefly summarise the effects of the remedy. Different people not only react surprisingly differently, but also in one and the same case the effects of the remedy vary from day to day without a satisfactory explanation of these results being forthcoming. In view of the capricious nature of the disease itself, and its varying intensity, not only in different cases but in the same patient at different times, this irregularity in action of the antitoxin is perhaps not surprising. Nevertheless it is so unsatisfactory that, for the present, Sir Felix Semon prefers to consider his views as "impressions" rather than as fixed opinions. He administered the remedy in all cases himself personally, and before the beginning of the hay fever season, he inquired from Professor Dunbar and Dr. Pransnitz whether it would not be right to try and ward off attacks altogether by subcutaneous use of the remedy. He was advised, however, not to adopt this course, since trials made in Hamburg had shown that subcutaneous injection of the antitoxin in some persons predisposed to hay fever had been followed by swelling of the injected limb accompanied by urticaria and erythema.

From this and a subsequent communication from Professor Dunbar it is obvious that the serum as at present prepared is not quite free from properties of a toxic nature. These it would appear from Professor Dunbar's observations are not essential to its therapeutic action, but are by-effects which probably to some extent interfere with the latter, and it is hoped that modification of the process of manufacture may eventually lead to their elimination. Such as they are these toxic effects are naturally more likely to result if the serum is used subcutaneously, and Sir Felix Semon therefore employed it by instillation only, and so used he observed no undesirable effects whatever. He considers, therefore, that it is a remedy which may be safely put into the hands of all reasonable patients, provided that they are warned not to use it too freely; for under such circumstances it is possible that the same œdematous and erytheatous conditions, observed after subcutaneous injection, might occur.

The preparations with which Sir Felix Semon made his trials were partly 1 in 500 antitoxin mixed by himself with an equal quantity of normal horse serum, and partly the ready-made preparation of the same sera which are being sold under the name of "Pollantin." The ready-made preparation, however, does not appear to be put upon the market in as suitable a form as it might be, for the author complains that the corks are bad and the pipettes supplied too short, so that in use a considerable portion of the serum is wasted. At its price of 10s. a case it is thought that errors of this sort might well be avoided by putting the remedy into receptacles more commodious and practical for a remedy the use of which has to be repeated at odd moments; that is to say, when sudden warnings of an on-coming attack are believed to be observed. The general directions given are to instil a drop of the serum inside the downdrawn eyelid, or three or four, followed by snuffing, inside the nostrils.

In dealing with his cases the author employed the remedy in some cases once, in others twice, and in others thrice a day. He summarises the impressions gathered from its use in this way by saying that, as might have been expected in an affection in which during the critical time of the year constant reinfection is liable to occur, he cannot consider the remedy truly curative in the sense of being one, a single application of which will ward off or cure the disease for any prolonged period. Whether it will be possible to modify the serum so as to abolish the unpleasant by-effects noted when used subcutaneously, and whether its use in this fashion will be more effective remains to be seen.

The remedy, however, would still be of advantage if by repeated application to the nose or eyes, attacks could be prevented for a limited period, or established attacks cut short. In some of his cases it did seem decidedly to have this effect, but it varied with all of them, and in none could it be counted on with certainty. Sometimes it acted and sometimes it did not. In fact, the only point in which constant benefit was observed in all cases was as regards the eyes, the irritability of which was immediately relieved. The duration, however, of this relief was variable. Sir Felix sums up by saying that the remedy certainly did not act as a panacea in any of his cases, although it gave a certain measure of relief in some and in others appeared to postpone attacks which would otherwise have occurred. More frequent use of the remedy might possibly punctuate these effects, and if with further experience it is found that increased frequency in use produces neither unpleasant effects nor diminishes the good ones, such as they are, the remedy may, perhaps, be considered a useful addition to the therapeutic means at command in the treatment of this disease.

[1] Brit. Med. Jour., July 18, 1903.

